# Food Starch Structure Impacts Gut Microbiome Composition

**DOI:** 10.1128/mSphere.00086-18

**Published:** 2018-05-16

**Authors:** Frederick J. Warren, Naoki M. Fukuma, Deirdre Mikkelsen, Bernadine M. Flanagan, Barbara A. Williams, Allan T. Lisle, Páraic Ó Cuív, Mark Morrison, Michael J. Gidley

**Affiliations:** a Centre for Nutrition and Food Sciences, Queensland Alliance for Agriculture and Food Innovation, The University of Queensland, St. Lucia, Brisbane, Queensland, Australia; b Diamantina Institute, Faculty of Medicine, The University of Queensland, Woolloongabba, Brisbane, Queensland, Australia; c Translational Research Institute, The University of Queensland, Woolloongabba, Brisbane, Queensland, Australia; d Research Faculty of Agriculture, Hokkaido University, Sapporo, Japan; e ARC Centre of Excellence in Plant Cell Walls, Centre for Nutrition and Food Sciences, Queensland Alliance for Agriculture and Food Innovation, The University of Queensland, St. Lucia, Brisbane, Queensland, Australia; f School of Agriculture and Food Sciences, The University of Queensland, Gatton, Queensland, Australia; University of Wisconsin—Madison

**Keywords:** carbohydrate structure, fermentation, microbial ecology, resistant starch, short-chain fatty acids

## Abstract

Starch is a major source of energy in the human diet and is consumed in diverse forms. Resistant starch (RS) escapes small intestinal digestion and is fermented in the colon by the resident microbiota, with beneficial impacts on colonic function and host health, but the impacts of the micro- and nanoscale structure of different physical forms of food starch on the broader microbial community have not been described previously. Here, we use a porcine *in vitro* fermentation model to establish that starch structure dramatically impacts microbiome composition, including the key amylolytic species, and markedly alters both digestion kinetics and fermentation outcomes. We show that three characteristic food forms of starch that survive digestion in the small intestine each give rise to substantial and distinct changes in the microbiome and in fermentation products. Our results highlight the complexity of starch fermentation processes and indicate that not all forms of RS in foods are degraded or fermented in the same way. This work points the way for the design of RS with tailored degradation by defined microbial communities, informed by an understanding of how substrate structure influences the gut microbiome, to improve nutritive value and/or health benefits.

**IMPORTANCE** Dietary starch is a major component in the human diet. A proportion of the starch in our diet escapes digestion in the small intestine and is fermented in the colon. In this study, we use a model of the colon, seeded with porcine feces, in which we investigate the fermentation of a variety of starches with structures typical of those found in foods. We show that the microbial community changes over time in our model colon are highly dependent on the structure of the substrate and how accessible the starch is to colonic microbes. These findings have important implications for how we classify starches reaching the colon and for the design of foods with improved nutritional properties.

## Abstract (precis)

Dietary starch is a major component in the human diet. A proportion of the starch in our diet escapes digestion in the small intestine and is fermented in the colon. In this study, we use a model of the colon, seeded with porcine feces, in which we investigate the fermentation of a variety of starches with structures typical of those found in foods. We show that the microbial community changes over time in our model colon are highly dependent on the structure of the substrate and how accessible the starch is to colonic microbes. These findings have important implications for how we classify starches reaching the colon and for the design of foods with improved nutritional properties.

## INTRODUCTION

Many gut bacteria are known to degrade and/or ferment starch (1). The starch uptake system (*sus*) of *Bacteroides thetaiotaomicron* serves as the progenote model of the polysaccharide utilization loci (PUL) across the *Bacteroidetes* phylum, and more recently, the “amylosome” multiprotein complexes of the Gram-positive *Firmicutes* bacterium Ruminococcus bromii have been characterized (2–4). Other commensal bacteria widely recognized for their beneficial effects on human health, such as Eubacterium rectale and Faecalibacterium prausnitzii can utilize the maltodextrins released by these degradative bacteria to support their growth (5–8).

Several structural features have been identified that can lead to starch escaping digestion in the small intestine (SI) and thereby being defined as resistant starch (RS) (5, 9, 10). These features include the following: (i) the native, semicrystalline (double-helical), granular form of starch, such as that found in raw foods, such as bananas; (ii) the partially recrystallized double-helical structures that form when starch is cooked and allowed to cool found, for example, in cold potatoes and stale bread; and (iii) starch which is encapsulated within matrices such as intact plant tissue or processed forms such as pasta, and therefore not available for digestion by small intestinal enzymes (11). It has been shown that starch from these materials is recovered from ileal effluents and therefore is available for fermentation in the colon (9, 12, 13).

In addition to our enhanced understanding of the role of specific gut microbiota, the positive effects from resistant starches on metabolic and cellular measures in human and animal studies have prompted a resurgent interest in their use to prevent and control digestive disorders and/or diseases, for example, improvements in insulin sensitivity which result from the effects of microbial metabolites produced during RS fermentation on secretion of gut peptides involved in appetite regulation and glucose homeostasis (14, 15). Furthermore, while it is known that starch structure can influence the ability of isolated bacterial species to ferment starch (5), there is limited understanding of how these properties affect temporal and/or spatial variations in the microbiome, as well as starch fermentation kinetics and characteristics.

Here, we examine how variations in food starch structure affect the gut microbiome, together with the kinetics of fermentation and the subsequent production of short-chain fatty acids (SCFA). We use a batch fermentation system inoculated with fecal microbiota from pigs fed a tightly controlled low-RS diet. This resulted in a fecal inoculum which was naive to RS prior to the start of the experiment, due to the low intake of RS in the pig’s diet. Pigs have long been used as a model for the human digestive tract, as they are omnivorous and have similar organ structures and functions, and importantly, the gut microbiota of pigs is relatively similar to that of humans (16, 17).

By utilizing a wide range of physical structures of starch substrates, we drive reproducible changes in microbial community composition and fermentation kinetics. The samples analyzed included examples of the following: (i) starch encapsulated within plant tissue (sorghum grain tissue [ST], maize kernel tissue [MT], potato tuber tissue [PT], chickpea endosperm tissue [CT]) or processed food (pasta [PA]); (ii) the native granular form of starch (potato with and without prior amylase treatment; native potato starch [PS] and native potato starch [amylase digested] [PSA]); (iii) partially recrystallized starches from cook/cool treatments (potato and high-amylose maize with and without prior amylase treatment; cooked and recrystallized potato starch [PSC], PSC [amylase digested] [PSCA], cooked and recrystallized maize starch [MSC], and MSC [amylase digested] [MSCA]). These three forms of starch are commonly referred to as RS types I, II, and III, respectively, in the literature (11). The findings demonstrate that different starch substrates produce defined temporal changes in microbiome composition during fermentation. Three distinct microbial communities were identified dependent on substrate structure, which resulted in distinct fermentation kinetics and SCFA production profiles. The substrates giving rise to the three microbial communities did not correspond to the conventional structural definitions of RS and suggest a new classification of RS based on microbiota response.

## RESULTS

### Starch structure and interactions with microbiota during fermentation.

**FIG 1:**
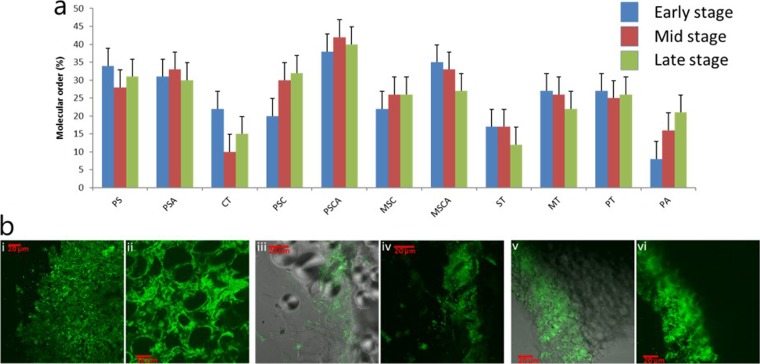
^13^C CP/MAS NMR analysis and microscopy of substrates. (a) Percentage double-helical order in starch substrates before fermentation (blue), during the exponential phase of fermentation (red) and at the endpoint of fermentation (green). (b) FISH visualization of the bacterial biofilms (green) associated with the nonstarch polysaccharide (NSP)-containing substrates pasta (i and ii), potato tissue (iii and iv), and maize tissue (v and vi) after 24 h of fermentation. Bacteria were hybridized with the EUB338Mix FITC probe.

We first examined the double-helical order present in the starch for each substrate, calculated from their ^13^C cross-polarization magic angle spinning (CP/MAS) nuclear magnetic resonance (NMR) spectra (18). There was large variation in the helical order at the start of fermentation between the different substrates. However, only PSC and PA showed a significant change in molecular order with fermentation time, indicating that microbial amylolytic enzymes are able to attack crystalline and amorphous starch equally efficiently (Fig. 1a). Fermentations coupled to fluorescence *in situ* hybridization (FISH) using the universal bacterial probe mix and confocal laser scanning microscopy enabled visualization of the biofilms associated with food and plant tissue particulate substrates PA, PT, and MT, respectively, after 24 h of fermentation (Fig. 1b). It is evident that the microarchitecture of these substrates influenced bacterial access to starch within these substrates at this fermentation time point. For PA, the starch within the outer matrix of the strands is readily degraded, with no granular morphologies maintained (Fig. 1bi), while in the center of the PA strands, bacterial cells surround and begin to envelop the intact starch granules (Fig. 1bii). For PT, those starch granules encased within the fine cell wall matrices remain relatively intact, and the bacteria are primarily associated with the cell wall components. However, those granules free of the cell wall matrices are extensively decorated with a biofilm (Fig. 1biii and 1biv). For MT, the cell wall matrices appear to be more rigid and thicker than observed for PT, thereby limiting bacterial access primarily to those granules at the peripheral edges of the kernel (Fig. 1bv and 1bvi).

### Temporal dynamics of microbiome composition during fermentation characterized by 16S rRNA gene amplicon sequencing.

**FIG 2:**
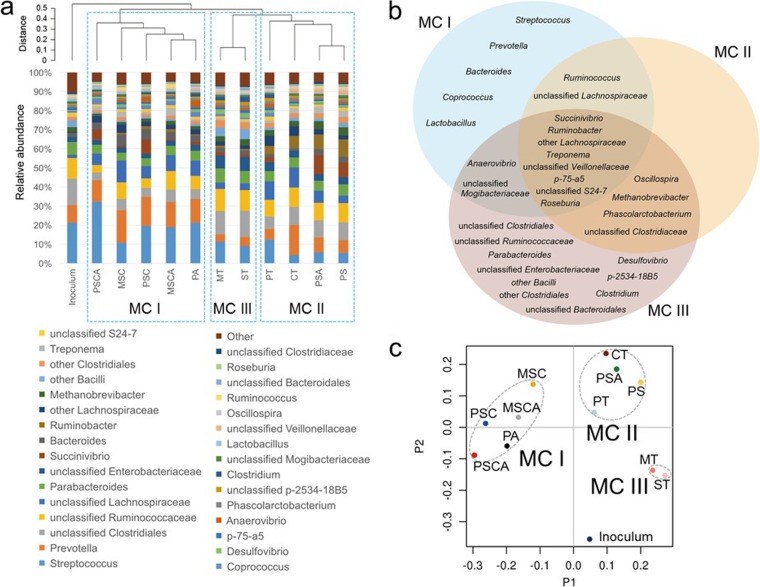
Starch substrates produce three distinct microbial community profiles during fermentation. (a) Compared to the starting inoculum, the communities could be subdivided into three microbial communities (MC; I, II, and III) based on hierarchical clustering of the genus distribution profiles produced from each substrate. (b) All three MCs share a relatively small core microbiome, and distinct genera can differentiate between the MC-I and MC-III profiles from MC-III (*P* < 0.05) abundance. (c) The PCoA plots of the communities produced for each substrate retained the overall clustering and were clearly separable from the starting inoculum. The substrate designations are the same as those described in the text.

We identified 31 genera that comprised ≥0.5% of the microbial communities across all substrates and fermentation time points (see Fig. S1 in the supplemental material). The distribution pattern of those genera was significantly different among the purified starches (Fig. S2) and tissue substrates (Fig. S3). The temporal distributions of these 31 genera throughout the fermentation time course were analyzed to generate a hierarchical clustering (Fig. 2a; see Data Set S1 for relative operational taxonomic unit [OTU] abundances). The 11 types of substrates clustered into three different microbial communities (MCs). Communities fermenting the cooked and cooled starches (PSC, PSCA, MSC, and MSCA) as well as PA clustered together and formed microbial community I (MC-I). MC-II comprised native potato starches (PS and PSA), CT, and PT. Microbial communities fermenting MT and ST clustered together and formed MC-III (Fig. 2a). The MC-I microbiome included higher abundance of well-characterized gut bacterial genera, including *Streptococcus*, *Prevotella*, *Bacteroides*, *Coprococcus*, and *Lactobacillus*, while MC-III included higher abundance of *Parabacteroides*, *Desulfovibrio*, *Clostridium*, and several unclassified bacterial groups compared with other MCs. The MC-II formed an intermediate community between MC-I and MC-III (Fig. 2b). This clustering was confirmed by principal-coordinate analysis (PCoA) plotting (Fig. 2c).

10.1128/mSphere.00086-18.1 FIG S1  Dynamics of microbial taxonomic distribution for each starch fermentation. Stream plots exhibiting those genera that comprise ≥0.5% of the overall microbial communities produced from each substrate. The substrate abbreviations are the same as those given in the text and are grouped in columns denoting MCI, MCII, and MCIII, respectively. Download FIG S1, DOCX file, 0.9 MB. Copyright © 2018 Warren et al. 2018 Warren et al. This content is distributed under the terms of the Creative Commons Attribution 4.0 International license.

10.1128/mSphere.00086-18.2 FIG S2  Key shifts in microbial genera comparing substrates with and without amylase treatment, native and cooked, cooled starches, and purified potato and maize starches. Microbial genera showing significant differences in mean proportions are shown, along with the mean proportions and 95% confidence intervals for each genus. Download FIG S2, DOCX file, 0.2 MB. Copyright © 2018 Warren et al. 2018 Warren et al. This content is distributed under the terms of the Creative Commons Attribution 4.0 International license.

10.1128/mSphere.00086-18.3 FIG S3  Key changes in microbial genera comparing different types of starch (chickpea, sorghum, maize tissue, and potato tissue) irrespective of pretreatment, as well as pasta. The box-and-whisker plots for each bacterial genus denote the first and third quartiles and maximum and minimum relative proportions, respectively. Significant differences between each starch type are denoted by different letters (*P* < 0.05). Download FIG S3, DOCX file, 0.2 MB. Copyright © 2018 Warren et al. 2018 Warren et al. This content is distributed under the terms of the Creative Commons Attribution 4.0 International license.

10.1128/mSphere.00086-18.8 DATA SET S1  Excel file containing the relative OTU abundancies used to produce the clustering analysis shown in Fig. 2a. Download DATA SET S1, XLSX file, 0.01 MB. Copyright © 2018 Warren et al. 2018 Warren et al. This content is distributed under the terms of the Creative Commons Attribution 4.0 International license.

**FIG 3:**
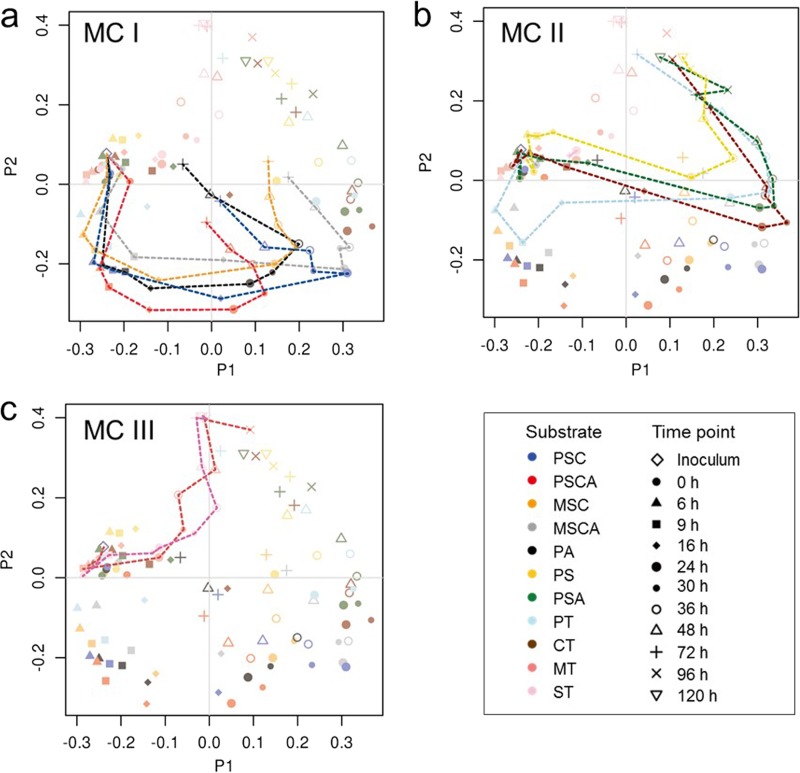
Microbial community dynamics for MC-I, MC-II, and MC-III are different. (a to c) The PCoA plots of the genus distribution profiles for individual samples collected at different time points during the fermentation of each substrate, with those substrates giving rise to the MC-I, MC-II, and MC-III profiles indicated by the dashed lines in panels a to c. Here, the profile of the starting inoculum is placed central to the samples collected from each substrate fermentation at 0 h, as indicated in each panel. Each substrate is color coded, and its designation is the same as those described in the text. Datum points for other substrates are included for reference.

These three MCs also showed differential kinetic shifts, represented by taxonomic distribution patterns as well as PCoA plottings (Fig. 3 and Fig. S1). MC-I showed rapid growth and predominance of *Streptococcus* in the first 6 h, resulting in depression of the community diversity, and both *Streptococcus* and *Prevotella* were the predominant genera throughout the fermentation. In MC-II, those two genera decreased by the middle of the fermentation period, with *Ruminobacter* gradually increasing and becoming dominant from 16 h to 30 h, followed by the dominance of *Succinivibrio* and/or unclassified *Lachnospiraceae* for the remainder of the fermentation. MC-III did not show a major community shift in the beginning and middle phases of fermentation, but after 48 h of incubation, unclassified *Clostridiales* and other *Bacillus* species became dominant groups, leading to a reduction in the diversity. PCoA based on a genus-level distribution showed that the microbiota composition from each of the MC groupings diverged throughout the time course of the experiment, from a similar initial condition (Fig. 3).

### Functional properties inherent to the microbiomes present in the different fermentations were predicted using PICRUSt.

**FIG 4:**
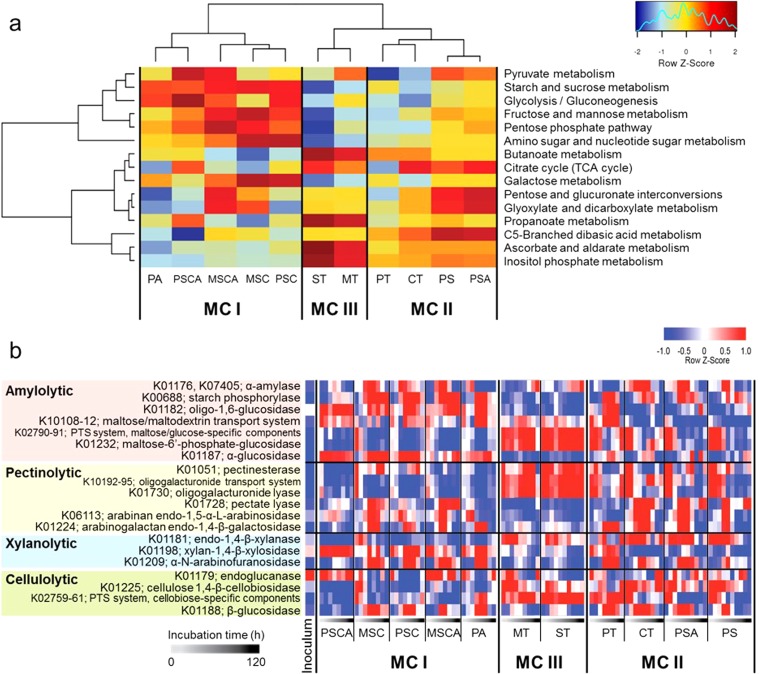
Dynamics of inferred gene abundance profiles for polysaccharide degradation and solute transport. Gene functions were predicted and quantified from the taxonomic profiles determined for the different substrate fermentations at each sampling time point using PICRUSt. (a) Distribution of genes present in pathways categorized in carbohydrate metabolism. TCA, tricarboxylic acid. (b) The heat map represents the Z-score change for each predicted gene relative to the mean abundance for each KEGG (Kyoto Encyclopedia of Genes and Genomes) gene group, calculated from all data combined.

Profiles of the predicted genes annotated for carbohydrate metabolism also clustered fermentation substrates into the same three MCs (Fig. 4a). Of these clusters, MC-I possessed the highest inferred gene counts annotated for starch and sucrose metabolism and polysaccharide degradation and metabolism. This indicates that the PA, PSC, PSCA, MSC, and MSCA starches promoted highly amylolytic microbes, including *Streptococcus*, *Prevotella*, and *Bacteroides* in MC-I (as shown above). The detailed enzymatic profiles for carbohydrate degradation and transportation were investigated from the output of the PICRUSt analysis (Fig. 4b). Over the length of time of incubation, the MC-I is predicted to possess a greater variety of the major amylolytic enzymes, such as α-amylase, starch phosphorylase, and oligo-1,6-glucosidase, and these were less abundant in MC-II- and MC-III-driven fermentations. This corresponds to the relatively accessible but densely packed starch in the cooked, cooled starch and the cooked pasta samples which give rise to MC-I. In contrast, a greater variety of pectinolytic, xylanolytic, and cellulolytic enzymes were putatively identified in MC-II and MC-III. The enzyme profile for MC-III did not show a drastic kinetic shift, which corresponds well to the kinetics of the taxonomic distribution (Fig. S1). Several enzymes in the MC-II profiles showed time-specific patterns, where enzymes active on polysaccharides (pectinesterase, endo-1,4-β-xylanase, and cellulose 1,4-β-cellobiosidase) were detected in the early to middle stage of incubation, while those active on the degradation products or polysaccharide side chains (arabinogalactan endo-1,4-β-galactosidase, α-*N*-arabinofuranosidase, and β-glucosidase) were found in the middle to late incubation times. These outcomes suggest that the MC-II gradually degraded the nonstarch polysaccharides (pectin, cellulose, and hemicellulose) in the highly hydrated potato and chickpea tissues, whereas MC-III slowly and minimally broke down MT and ST starches encapsulated within the much more densely packed (less-hydrated) cereal grain tissues. Thus, the early stages of MC-II development followed those of MC-III because both involve cell wall degradation, with later stages of MC-II development being similar to MC-I where starch degradation predominates. The interesting nonconformers in this analysis are the native potato starch (with or without prior amylase treatment), which clustered with chickpea and potato tissue in MC-II, and the cooked, cooled potato starch (with or without prior amylase treatment) in MC-I. It may be that this reflects the presence of species specialized in degrading the incompletely gelatinized starch in the potato and chickpea tissue, rather than the presence of cell wall degraders.

### Gas production kinetics and SCFA production directly relate to microbial community composition.

For both the maximum rate of gas production (*R*_max_) and the time at which this occurs (*T*_*R*max_), significant differences were observed between the different MC groupings, indicating that changing the physical structure of the substrate directly influenced fermentation behavior. MC-I and MC-II had more closely related gas production kinetics, with no significant difference in the overall half-time (*T*_1/2_) of gas production, while MC-III showed slower fermentation and statistically different values for all gas kinetic parameters (Table 1). This reflects the microbial community composition results (Fig. 2a and c), showing that MC-II shared closer community characteristics with MC-I than MC-III. As expected, acetate was the main SCFA produced for all substrates, followed by propionate and smaller amounts of butyrate, with differences seen in their relative proportions, and that of the ratio of branched-chain to linear SCFA, depending on the substrate and MC (Table 2).

**TABLE 1:** Kinetic parameters for gas production vary depending on substrate and microbial community^a^

Microbial community and substrate or parameter^b^	DMCV (ml)^c^	*T*_*R*max_ (h)	*R*_max_ (ml/h)	*T*_1/2_ (h)
MC-I	**349 (4.2) A**	**19.5 (0.90) A**	**9.6 (0.19) A**	**31.9 (1.79) A**
PSC	380 AB	17.3 CDE	11.4 AB	25.6 DE
PSCA	385 A	8.78 E	12.1 A	23.3 E
MSC	326 C	26.8 ABC	8.0 CDE	36.8 CDE
MSCA	340 BC	24.1 ABC	7.2 DE	45.8 C
PA	312 C	20.9 BCD	9.5 BC	28.1 CDE
MC-II	**331 (4.6) B**	**27.8 (0.98) B**	**7.7 (0.21) B**	**36.6 (1.96) A**
PS	312 C	32.9 A	5.9 E	43.1 CD
PSA	375 AB	29.4 AB	7.8 CDE	38.7 CDE
PT	311 C	19.4 CD	9.0 CD	28.2 CDE
CT	324 C	29.5 AB	8.0 CD	36. CDE
MC-III	**218 (6.4) C**	**10.6 (1.39) C**	**2.7 (0.30) C**	**98.4 (2.77) B**
MT	200 D	9.3 E	2.3 F	131.1 A
ST	236 D	11.9 DE	3.2 F	65.7 B
Prob-MC	<0.0001	<0.0001	<0.0001	<0.0001
Prob-sub(MC)	<0.0001	<0.0001	<0.0001	<0.0001
MSD*	44.3	9.6	2.09	19.05

a The substrates are purified starch substrates, analyzed by botanical origin, hydrothermal pretreatment, and α-amylase pretreatment, and substrates containing nonstarch components. Values indicated with different letters are significantly different (*P* < 0.05). The values for microbial communities I, II, and III are shown in boldface type.

b Prob-MC, probability—microbial community; Prob-sub(MC), probability—substrate (microbial community); MSD*, maximum standard deviation.

c DMCV, dry matter cumulative gas production.

**TABLE 2:** SCFA and ammonia production are dependent on substrate and microbial community composition^a^

Microbial community and substrate or parameter	Concn (mmol/g [DM])					% AcTot	% PrTot	% BuTot	% BrChPpn
	Acetic acid	Propionic acid	Butyric acid	Total SCFA	NH_3_				
MC-I	**5.6 (0.071) A**	**3.7 (0.56) A**	**0.20 (0.003) B**	**11.8 (0.14) A**	**3.6 (0.05) B**	**47.4 (0.11) B**	**31.7 (0.21) A**	**1.7 (0.03) C**	**0.203 (0.002) C**
PSC	5.6 BC	4.3 AB	0.20 ABC	12.3 B	3.9 BC	45.2 F	35.1 A	1.6 EF	0.170 AB
PSCA	7.5 A	4.6 A	0.19 ABC	14.3 A	4.1 BCD	52.5 A	32.2 BC	1.4 F	0.149 A
MSC	5.3 BCD	3.4 DE	0.21 AB	11.2 BC	4.3 CD	47.3 DE	29.9 CD	1.9 CDE	0.202 CD
MSCA	5.3 BCD	3.4 CDE	0.21 AB	10.6 BCD	3.9 B	49.6 C	32.1 BC	2.0 CD	0.180 ABC
PA	4.4 DEF	3.0 EF	0.17 C	10.2 CD	2.0 A	42.7 G	29.3 D	1.7 DE	0.313 F
MC-II	**4.9 (0.11) B**	**3.4 (0.89) B**	**0.21 (0.005) A**	**10.5 (0.22) B**	**3.9 (0.08) A**	**46.5 (0.18) C**	**32.5 (0.34) A**	**2.0 (0.04) B**	**0.220 (0.004) B**
PS	4.5 DEF	3.1 CDEF	0.22 A	9.9 CD	4.6 D	45.5 F	33.2 AB	2.3 BC	0.198 BCD
PSA	6.2 B	4.2 ABC	0.21 ABC	12.6 AB	4.1 BCD	48.9 CD	33.2 AB	1.6 DEF	0.179 ABC
PT	4.1 EF	2.7 F	0.19 ABC	8.9 D	1.9 A	45.9 F	30.5 BCD	2.1 C	0.268 E
CT	4.9 BCD	3.5 BCD	0.23 A	10.7 BCD	4.8 D	45.7 EF	33.1 AB	2.1 BC	0.234 D
MC-III	**3.2 (0.18) C**	**1.5 (0.14) C**	**0.17 (0.007) C**	**6.3 (0.34) C**	**4.0 (0.12) A**	**51.0 (0.27) A**	**23.2 (0.52) B**	**2.6 (0.06) A**	**0.330 (0.005) A**
MT	3.0 FG	1.4 E	0.16 C	5.9 E	4.0 BCD	51.6 AB	23.5 E	2.7 A	0.377 G
ST	3.4 FG	1.5 E	0.17 BC	6.7 E	3.9 BCD	50.5 BC	22.8 E	2.5 AB	0.283 EF
Prob-MC	<0.0001	<0.0001	0.0003	<0.0001	0.0001	<0.0001	<0.0001	<0.0001	<0.0001
Prob-sub(MC)	<0.0001	<0.0001	0.0011	<0.0001	<0.0001	<0.0001	<0.0001	<0.0001	<0.0001

a The SCFA and ammonia products were analyzed by botanical origin, hydrothermal pretreatment and α-amylase pretreatment, and for substrates containing nonstarch components. Values indicated with different letters are significantly different (*P* < 0.05). The values for microbial communities I, II, and III are shown in boldface type. Abbreviations: DM, dry matter; AcTot, acetic acid total; PrTot, propionic acid total; BuTot, butyric acid total; BrChPpn, branched-chain fatty acid proportion; Prob-MC, probability—microbial community; Prob-sub(MC), probability—substrate (microbial community).

In MC-I and MC-II, a tight network was observed among *Streptococcus*, *Prevotella*, *Lactobacillus*, and *Bacteroides* (Fig. S4 and S5). For MC-III (Fig. S6), the companion consortia were strikingly different and included unclassified bacterial groups (*Clostridiales*, *Veillonellaceae*, and *Bacteroidales*) and strongly favored fermentation schemes resulting in acetate and butyrate, reflected in the significantly higher production of acetate and butyrate during the fermentation of MC-III substrates. In addition, for MC-III, there was an increased production of branched-chain fatty acids (Table 2), suggesting greater protein fermentation. Consistent with this, the starch in these fermentations was less degradable as judged by the lower SCFA levels.

10.1128/mSphere.00086-18.4 FIG S4  Microbial network and its correlation to SCFA production in MCI. Red, acetate; green, propionate; blue, butyrate; brown, BCR. Download FIG S4, DOCX file, 0.3 MB. Copyright © 2018 Warren et al. 2018 Warren et al. This content is distributed under the terms of the Creative Commons Attribution 4.0 International license.

10.1128/mSphere.00086-18.5 FIG S5  Microbial network and its correlation to SCFA production in MCII. Red, acetate; green, propionate; blue, butyrate; brown, BCR. Download FIG S5, DOCX file, 0.3 MB. Copyright © 2018 Warren et al. 2018 Warren et al. This content is distributed under the terms of the Creative Commons Attribution 4.0 International license.

10.1128/mSphere.00086-18.6 FIG S6  Microbial network and its correlation to SCFA production in MCIII. Red, acetate; green, propionate; blue, butyrate; brown, BCR. Download FIG S6, DOCX file, 0.3 MB. Copyright © 2018 Warren et al. 2018 Warren et al. This content is distributed under the terms of the Creative Commons Attribution 4.0 International license.

### Amylolytic activity can be identified in the cell-free medium.

An increase in amylolytic activity during fermentation was found in the spent cell-free medium for several substrates, indicating the release of amylolytic enzymes from the microbiota as the starch was fermented (Fig. S7a to d). To explore whether the same structural features that limit small intestinal α-amylase digestion also affect microbial digestion kinetics, *in vitro* digestion experiments of starches with and without α-amylase pretreatment were carried out (Fig. S7e and f). While the starches more slowly digested by α-amylase were among those more slowly fermented by the microbiota, the trend was not statistically significant (*r*^2^ = 0.319; *P* = 0.113), suggesting that different factors are limiting to gut microbiota fermentation of starch than small intestinal fermentation; for example, the microbiota can degrade cell walls which would inhibit starch digestion in the small intestine, and the microbiota possess a wider range of amylolytic enzymes, which may be better able to degrade more crystalline starch. Evidence for this is also shown in Fig. 1a, which shows that crystalline and amorphous starch was degraded at the same rate during fermentation.

10.1128/mSphere.00086-18.7 FIG S7  Examples of gas profiles showing cumulative gas on a dry matter basis (DMCV) over time (green dots) with a monophasic exponential fit (orange line) with cumulative amylolytic activity overlaid (black dots). (a to d) Native potato starch (a), sorghum tissue (b), cooked-retrograded maize starch (c), and chickpea (d). (e and f) The relationship between starch digestion rate *k* (minute^−1^) and maximal fermentation rate *R*_max_ (in milliliters per hour) is shown in panel e, with kinetic parameters for *in vitro* starch digestion shown in panel f. Download FIG S7, DOCX file, 0.1 MB. Copyright © 2018 Warren et al. 2018 Warren et al. This content is distributed under the terms of the Creative Commons Attribution 4.0 International license.

## DISCUSSION

Identifying differences in microbial community composition linked to differences in end products from the fermentation of starches with different physical structures is a key step toward the rational design of foods containing resistant starches to provide targeted health benefits. Our results indicate that microbial community composition is linked to substrate structure (Fig. 2) (5, 19–22). This variability can be explored and understood only through the use of starches with highly characterized structures, representing a broad range of different physical forms of starch, differing in structure at the macro-, micro-, and nanolength scales (23, 24). Given the large differences in fermentation patterns observed in the present study, we can also expect that large differences exist when starch is fermented by the human gut microbiota (both *in vitro* and *in vivo*). Our findings reveal how starch structure not only affects the kinetics of starch fermentation but also the structure-function relationships and ecological succession of the microbiome that develops during this process, reflecting changes in microbiome composition observed in *in vivo* feeding experiments in both humans and swine (5, 24, 25).

Defined changes in microbial community in swine fed resistant starch have been observed in a number of feeding studies in pigs. Umu et al. fed pigs retrograded starch and observed increases in *Prevotella*, *Lachnospiraceae*, and *Ruminococcus*, consistent with our findings for MC-I starches (including all retrograded starch substrates) (24). Sun et al. fed pigs raw potato starch (equivalent to PS, an MC-II starch), and described a decrease in *Clostridium*, *Treponema*, *Oscillospira*, *Phascolarctobacterium*, RC9, and S24-7 and an increase in *Turicibacter*, *Blautia*, *Ruminococcus*, *Coprococcus*, and *Marvinbryantia* (26, 27). Many of these OTUs were also observed to show population shifts in MC-II in the *in vitro* experiments shown in this paper, for example *Clostridium*, *Trepenoma*, *Oscillospira*, *Phascolarctobacterium*, S24-7, and *Ruminococcus*. The time course results available using *in vitro* models add valuable additional information, for example showing that *Clostridium*, S24-7, and *Ruminococcus* are prevalent during the early stages of starch fermentation, but populations then drop during later stages of fermentation, when samples would be taken for *in vivo* experiments. The results shown here identify many similar OTUs to those seen *in vivo* but provide more detailed time course data and allow analysis across a wider range of substrates.

Identifying how different starches are fermented will allow for the design of foods containing resistant starch that drive potentially beneficial changes in gut microbiota (28, 29). It will also allow the identification of potentially less favorable forms of resistant starch. For instance, highly active amylolytic bacteria, such as Bacteroides vulgatus and Streptococcus bovis, and species whose populations have been shown to increase in response to diets rich in RS, such as Prevotella copri and Ruminococcus gnavus, are recognized to be associated with colorectal neoplasia (30), new onset rheumatoid arthritis (31), and pouchitis following colectomy in ulcerative colitis (UC) patients (32). In that context, many forms of colorectal cancer and ulcerative colitis principally arise in the distal colon and/or rectum, at which sites the microbiota may have been “preset” by the structural properties of the starch entering the large bowel. The large bowel operates much like a plug-flow digester, and the limited mixing of material means the resident microbiome that colonizes and matures with the food matrix does so under batch or fed-batch conditions (33). For these reasons, a time course assessment of the microbiome changes during batch fermentations, as conducted in the present study, offers insights not readily achievable from the small animal and clinical studies performed with different starches and/or foods thus far (7). The present work based on 16S rRNA sequencing is able to identify clear differences at the genus level during the time course of starch fermentation. Future work should aim to identify whether specific species with known disease associations are selected for by starches with defined structures to identify potential disease associations.

Our work highlights how the structural properties of the substrate can drive functional differences across the microbiome and points the way for the design of starch-containing foods with defined microbial communities and fermentation characteristics, as well as suggesting novel approaches to classifying RS on the functional basis of its fermentation pathway by the colonic microbiota.

## MATERIALS AND METHODS

### Substrates.

Native potato starch (catalog no. S4251; Sigma-Aldrich, St. Louis, MO) and Gelose 80 high-amylose maize starch (Penford Australia Ltd., Sydney, Australia) were obtained commercially. Maize kernels (cv. Pioneer) were a gift from Glen Fox (University of Queensland, Brisbane, Australia), white sorghum kernels (cv. QL12) were a gift from Ian Godwin (University of Queensland, Brisbane, Australia), and separated chickpea cells were a gift from Sushil Dhital (University of Queensland, Brisbane, Australia). Potatoes (cv. Mozart) and pasta (Barilla no. 5; Barilla S.p.A, Parma, Italy) were purchased from a local supermarket (Brisbane, Australia).

From the native potato starch and maize starch, cooked and cooled starches were prepared by boiling 25 g of starch in 250 ml of deionized water (dH_2_O) for 20 min. This was then cooled and stored in a fridge for 24 h to allow (partial) recrystallization. The resultant gel was then frozen at −80°C before freeze drying and grinding to a powder to produce cooked, cooled potato starch and maize starch, respectively. α-Amylase predigestion was conducted on selected substrates; native potato starch, cooked and cooled potato starch, and cooked and cooled maize starch following a modification of the INFOGEST protocol (34). Briefly, 7 g of starch was dispersed in 70 ml of phosphate-buffered saline (PBS) (catalog no. P4417; Sigma-Aldrich) containing 200 U/ml of porcine pancreatic α-amylase (E-PANAA; Megazyme, Bray, Ireland) and incubated at 37°C for 2 h with regular mixing by inversion. After 2 h, the reaction was stopped immediately by the addition of an equal volume of 0.3 M Na_2_CO_3_ (35). Samples were then centrifuged (2,000 × *g*, 5 min) and washed in dH_2_O three times, before washing the samples with ethanol and drying them by rotary evaporation.

Maize and sorghum kernels were prepared by soaking the kernels in excess dH_2_O overnight before rinsing and boiling in dH_2_O (20 g of kernel per 100 ml of water) for 1 h. After cooking, the kernels were halved (sorghum) or quartered (maize). Potato was prepared by cutting the potato into 5-mm cubes and cooking the potato cubes in dH_2_O (20 g in 200 ml) at 70°C for 5 min to achieve starch gelatinization while retaining intact cell walls (36). Pasta was prepared following the manufacturer’s instructions. The pasta was broken into 1-cm lengths and boiled in dH_2_O (20 g of pasta to 200 ml of water) for 8 min.

Dry matter content for the substrates was determined by oven drying in preweighed porcelain crucibles at 105°C for 24 h. Total starch content was determined using the Megazyme total starch assay (K-TSTA; Megazyme, Bray, Ireland). All substrates were prepared freshly and weighed in clean serum bottles. Native potato starch, amylase-treated native potato starch, cooked and cooled potato and maize starches, and amylase-treated, cooked, cooled maize and potato starches had 0.2 g (dry weight) weighed into 50-ml fermentation bottles. Maize and sorghum kernels, chickpeas, pasta, and potato had 0.5 g (dry weight) weighed in 100-ml fermentation bottles. All the fresh cooked samples (maize and sorghum kernels, chickpeas, pasta, and potato) were weighed out 24 h prior to the start of the experiment and kept refrigerated, with regular CO_2_ sparging.

### *In vitro* starch digestion kinetics.

Substrates (100 mg [dry weight]) were weighed in 15-ml polypropylene tubes, to which 10 ml of phosphate-buffered saline (pH 7.2) was added and incubated at 37°C. At 0 min, porcine pancreatic α-amylase (E-PANAA; Megazyme, Bray, Ireland) was added to give 200-U/ml enzyme activity. Aliquots (100-µl aliquots) were taken at defined time points up to 180 min and added to 100 µl of 0.3 M Na_2_CO_3_ to stop the reaction. Reducing sugar production was measured using the 4-hydroxybenzoic acid hydrazide (PAHBAH) method of Lever (37) as described by Moretti and Thorson (38), using maltose standards. Absorbance was read using a Fluostar Optima plate reader (BMG Labtech, Mornington, Australia) at a wavelength of 405 nm. Kinetic parameters were determined using the logarithm of slope (LOS) method (39, 40).

### Collection and preparation of the inoculum.

Porcine feces were collected from five Large White grower pigs of 30 to 40 kg. The pigs had been fed a semipurified diet consisting mainly of rapidly digestible maize starch and fishmeal for 10 days prior to fecal collection. This diet was formulated to contain low levels of nonstarch polysaccharide, and a highly digestible starch that is rapidly and completely digested in the small intestine (SI), avoiding possible adaptation of the large intestinal microbiota to any of the substrates tested. Feces were collected per rectum with a gloved finger and placed immediately into a warmed vacuum flask previously flushed with CO_2_. The feces from all animals were combined and diluted 1:5 with prewarmed, sterile saline (0.9% NaCl). This mixture was mixed for 60 s using a handheld blender, and filtered through four layers of muslin cloth. All procedures were carried out under a constant stream of CO_2_.

### Batch fermentation and sampling.

Fermentation experiments were performed by the method of Williams et al. (41). Briefly, samples were inoculated with 2.5 ml (50-ml fermentation bottles) or 5 ml (100-ml fermentation bottles) of diluted and homogenized pig fecal inoculum, and the resulting samples were fermented at 39°C for up to 146 h (depending on the fermentation rate of the substrate). At a series of time points throughout fermentation, duplicate bottles were plunged into an ice-water bath for 20 min to halt bacterial activity and then opened for sampling. After the pH of the fermentation fluid was measured, samples were taken for short-chain fatty acid (SCFA) and NH_3_ analyses, as well as 500 µl of fluid for analysis of amylase activity, which was immediately frozen in liquid nitrogen (LN_2_). The remaining fermentation fluid was then centrifuged in a refrigerated centrifuge (4°C, 2,500 × *g*, 5 min), and samples of biomass were taken and frozen in LN_2_ for DNA extraction. The remaining biomass was frozen at −20°C and freeze-dried for ^13^C cross-polarization magic angle spinning (CP/MAS) nuclear magnetic resonance (NMR) analysis. Up to 25 gas readings were taken over a period of up to 146 h. Five replicates were used for determining gas kinetics. Substrate-free blanks were also prepared in duplicate for both the large and small bottles.

### ^13^C CP/MAS NMR.

The freeze-dried fermentation biomass from different time points was studied using solid-state ^13^C CP/MAS NMR at a ^13^C frequency of 75.46 MHz on a Bruker MSL-300 spectrometer (Bruker, Billerica, MA, USA). The powder was packed in 4-mm-diameter, cylindrical, PSZ (partially stabilized zirconium-oxide) tubes with a KelF endcap. The rotor was spun at 5 kHz at the magic angle (54.7°). The 90° pulse width was 5 µs, and a contact time of 1 ms was used for all samples with a recycle delay of 3 s. Other parameters were set as follows: spectral width, 38 kHz; acquisition time, 50 ms; time domain points, 2,000; transform size, 4,000; line broadening, 50 Hz. At least 2,400 scans were accumulated for each spectrum. Spectra were referenced to external adamantane. Molecular order values were obtained through fitting to a published multivariate analysis tool (18).

### FISH.

After 24 h of fermentation, residual potato tuber tissue (PT), pasta (PA), and maize kernel tissue (MT) substrates were handled with forceps, gently placed into 5 ml of prealiquoted 4% paraformaldehyde (PFA) fixative, and incubated at 4°C overnight. Thereafter, the PFA fixative was discarded, and the samples were washed to remove any residual fixative. The fixed samples were stored in equal volumes of 1× PBS and 100% ethanol solution at −20°C until processed.

Prior to fluorescence *in situ* hybridization (FISH), samples were sectioned using a Cryostat Microm HM5650 instrument to prepare 50-µm-thick sections. These sections were placed on slides which were coated with agarose to prevent loss of sample during hybridization. The slides were dehydrated and FISH was performed by the method of Gorham et al. (42). The universal bacterial probes EUB338, Eub338 II, and EUB338 III, labeled with fluorescein isothiocyanate (FITC), were combined as previously described (43) to form an equimolar mix for subsequent use.

### DNA extraction and sequencing.

Biomass from the 500-µl aliquots of each fermentation sample was concentrated by centrifugation (16,000 × *g*, 4°C, 5 min), and lysed by the RBB + C method (44). Total genomic DNA was extracted and purified using the Maxwell 16 LEV blood DNA kit (Promega, Madison, WI) and the Maxwell 16 MDx research instrument (Promega, Madison, WI) according to the manufacturer’s instructions. The concentration and purity of genomic DNA were evaluated with the NanoDrop Lite spectrophotometer (Thermo Fisher Scientific, Waltham, MA). The variable regions V6 to V8 of the 16S rRNA gene were amplified from the purified DNA. The primers used in this study consisted of the Illumina primer overhang adapters and bacterial universal primers as follows: the forward overhang adapter and primer 926F (5′-TCG TCG GCA GCG TCA GAT GTG TAT AAG AGA CAG AAA CTY AAA KGA ATT GRC GG-3′) and the reverse overhang adapter and primer 1392R (5′-GTC TCG TGG GCT CGG AGA TGT GTA TAA GAG ACA GAC GGG CGG TGW GTR C-3′). Dual-index barcodes were added to the amplicon target using the Nextera XT Index kit (Illumina, San Diego, CA). The concentrations of PCR product were measured by a QuantiFluor double-stranded DNA (dsDNA) system (Promega, Madison, WI), and all PCR products were pooled into one tube in equal amounts. Paired-end sequencing was performed using the Illumina MiSeq platform (Illumina, San Diego, CA), supported and operated by the Australian Centre for Ecogenomics (Brisbane, Australia).

### Analysis of 16S rRNA gene sequences.

The raw 16S rRNA gene sequence data were analyzed by Quantitative Insight Into Microbial Ecology (QIIME) version 1.9.1 (45). Sequences with a Phred score of lower than 20 were removed. Chimeric sequences were checked and removed using USEARCH version 6.1.544 (46). The remaining high-quality reads were clustered into operational taxonomic units (OTUs) by PyNAST (47) with a 97% sequence identity threshold against the Greengenes core set database version 13.8 (48). The generated biome table was normalized using an equal subsampling size of 2,938 sequences. Distances between bacterial communities in different samples were calculated by the weighted UniFrac distance metric (49) in QIIME. Calypso (50) version 5.2 was used to generate hierarchical clustering, principal-coordinate analysis (PCoA) plots, and microbial network maps. Metagenomic function was predicted using PICRUSt (51) online Galaxy version by the method of Umu et al. (24). Heat maps were generated using Calypso and gplots package in R.

The White’s nonparametric *t* test and Kruskal-Wallis H test were performed to compare the relative abundance of genera for two groups and more than three groups, respectively. Statistical significance was analyzed by Prism version 6.0g (GraphPad Software, La Jolla, CA). A *P* value of less than 0.05 was regarded as statistically significant.

### Gas production kinetic analysis.

Gas production kinetics were modelled using a monophasic exponential model as follows (52):

$$G=A/\left[1{\left(C/t\right)}^{B}\right]$$

where *G* is cumulative gas produced (in milliliters) at time *t* (in hours), *A* is asymptotic gas production, *B* is the switching characteristic of the curve, *C* is the time at which half of the asymptotic value has been reached (in hours), and *t* is time (in hours). Model parameters were estimated for each bottle, by nonlinear least squares with the SAS (version 9.3) NLIN procedure.

Two further parameters may be calculated from the results of this fitted curve, *R*_max_, which is the maximum rate of gas production; and *T*_*R*max_, the time at which *R*_max_ occurs:

$$R_{\max}=\frac{\left(A)(C^{B})(B)(T_{R\text{max}}{}^{-B-1}\right)}{{\left[1+(C^{B})(T_{R\text{max}}{}^{-B})\right]}^{2}}$$

$$T_{R\text{max}}=C{\left(\frac{B-1}{B+1}\right)}^{\frac{1}{B}}$$

For statistical analysis, the substrates were separated into two groups and analyzed separately. Group 1 contained complex substrates (chickpea cells, sorghum tissue, maize tissue, potato tissue, and pasta), and group 2 contained purified starches (native potato starch, cooked and cooled potato starch, and cooked and cooled maize starch with or without amylase treatment). Purified starches were further subdivided into cooked versus native, amylase-treated starch versus non-amylase-treated starch, and maize versus potato to identify systematic variations across the different substrates.

Analysis of variance was conducted on the gas production parameters using the GLM procedure (SAS 9.3 for Windows, Cary, NC, USA) with model terms for microbial community (MC) group and substrates nested within each group. Any significant differences between substrates and MC group as described above were tested using Tukey’s studentized range test.

### Measurement of fermentation products and amylolytic activity.

SCFAs were analyzed by gas chromatography using a Shimadzu GC-17A instrument (Kyoto, Japan), fitted with a ZB-FFAP column (30 m by 0.53 mm) (J & W Scientific, USA). The temperatures were as follows: 180°C for the injector, 210°C for the detector, and 85°C for the column. The temperature was held initially for 4 min and then increased at 15°C/min until the temperature reached 200°C, which was held for 2 min. The carrier gas was helium at 5.0 ml/min, 67 kPa for 2 min, and then 1.8 kPa/min to 81 kPa. The internal standard was 4-methyl valeric acid. Using the SCFA concentrations, the branched-chain ratio (BCR) was calculated. This is the ratio of mainly branched-chain acids (including valeric acid) to the straight-chain acids. The former is associated with the metabolism of amino acids, and the latter is associated with the metabolism of carbohydrates. Samples were analyzed for SCFA concentrations at time points throughout the fermentation and also at time zero. The SCFA values at time zero (contributed from the starting medium and inoculum) were taken into account in the subsequent analysis.

Ammonia was analyzed using a method modified from reference 53. Briefly, ammonium was determined colorimetrically, utilizing the chemical reaction of ammonium ions (NH_4_^+^) with sodium salicylate and nitroprusside in a weakly alkaline buffer, at a wavelength of 650 nm, using a high-throughput sample analyzer (AU400 chemistry analyzer; Olympus, Tokyo, Japan).

Amylolytic activity of the fermentation fluid was determined following filtration through a 0.2-µm syringe filter using the Enzcheck Ultra α-amylase assay (Life Technologies, Mulgrave, Australia) as directed by the manufacturer. Fluorescence was measured using a Fluostar Optima plate reader (BMG Labtech, Mornington, Australia) with an excitation wavelength of 460 nm and emission wavelength of 520 nm.

### Data availability.

Nucleotide sequence data reported in this study are available in the DDBJ Sequence Read Archive under the accession number DRA006773.
