# Supplementary figures and images for: Food Starch Structure Impacts Gut Microbiome Composition
Source: mSphere. 2018 May 16;3(3):e00086-18. doi: 10.1128/mSphere.00086-18 (PMC5956147; doi:10.1128/mSphere.00086-18)

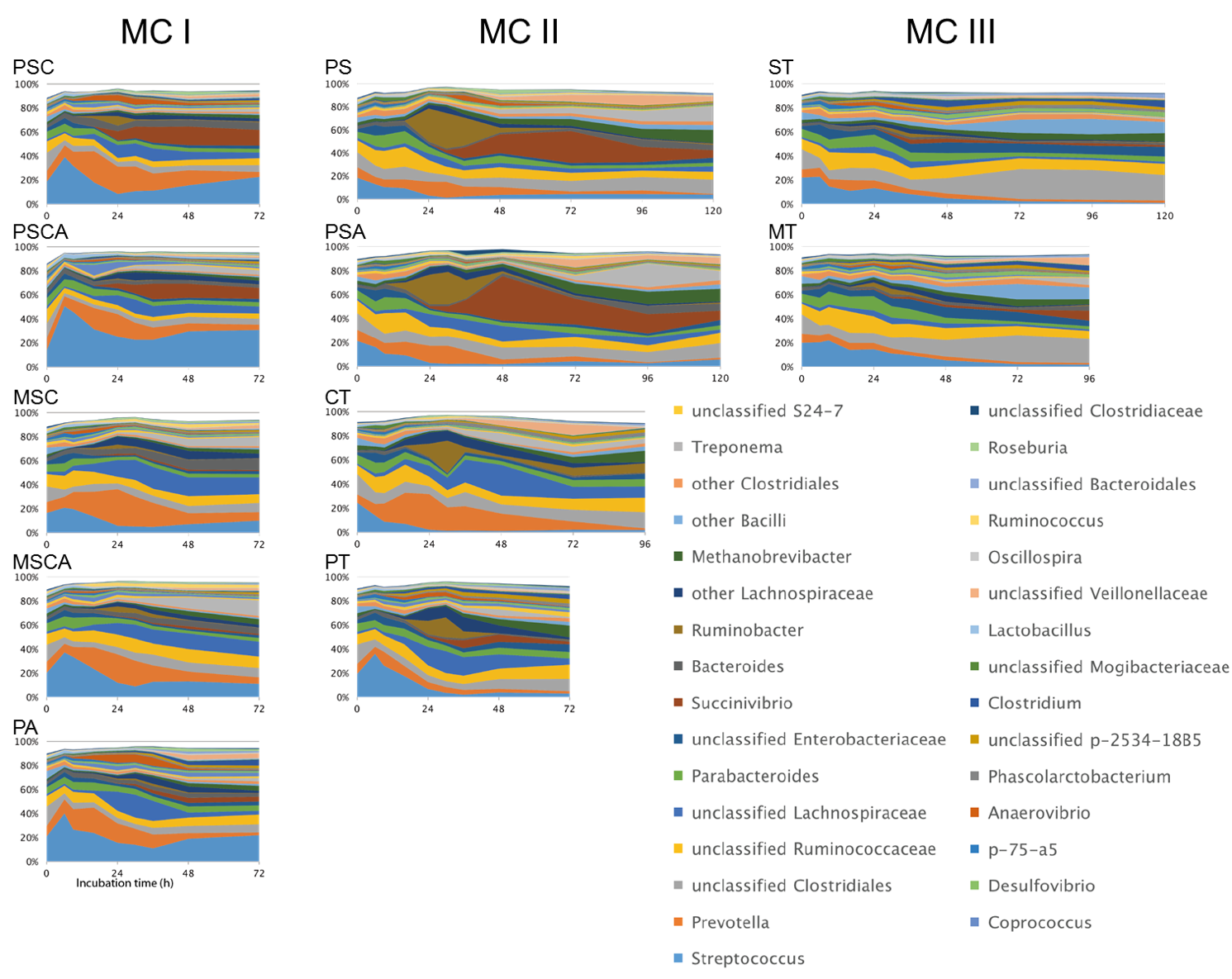


**Figure S1**

Supplement: FIG S1 [file sph003182543sf1.docx]

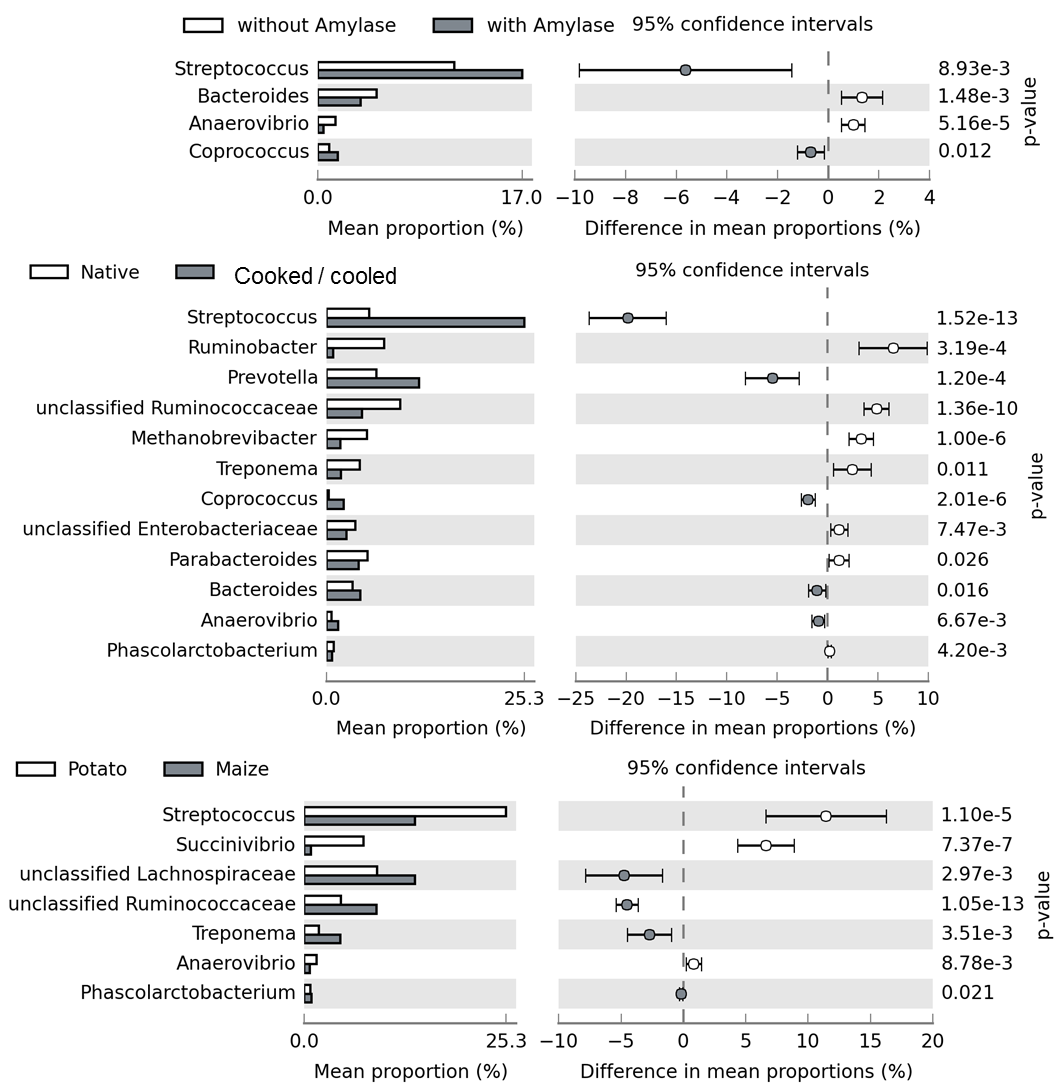


**Figure S2**

Supplement: FIG S2 [file sph003182543sf2.docx]

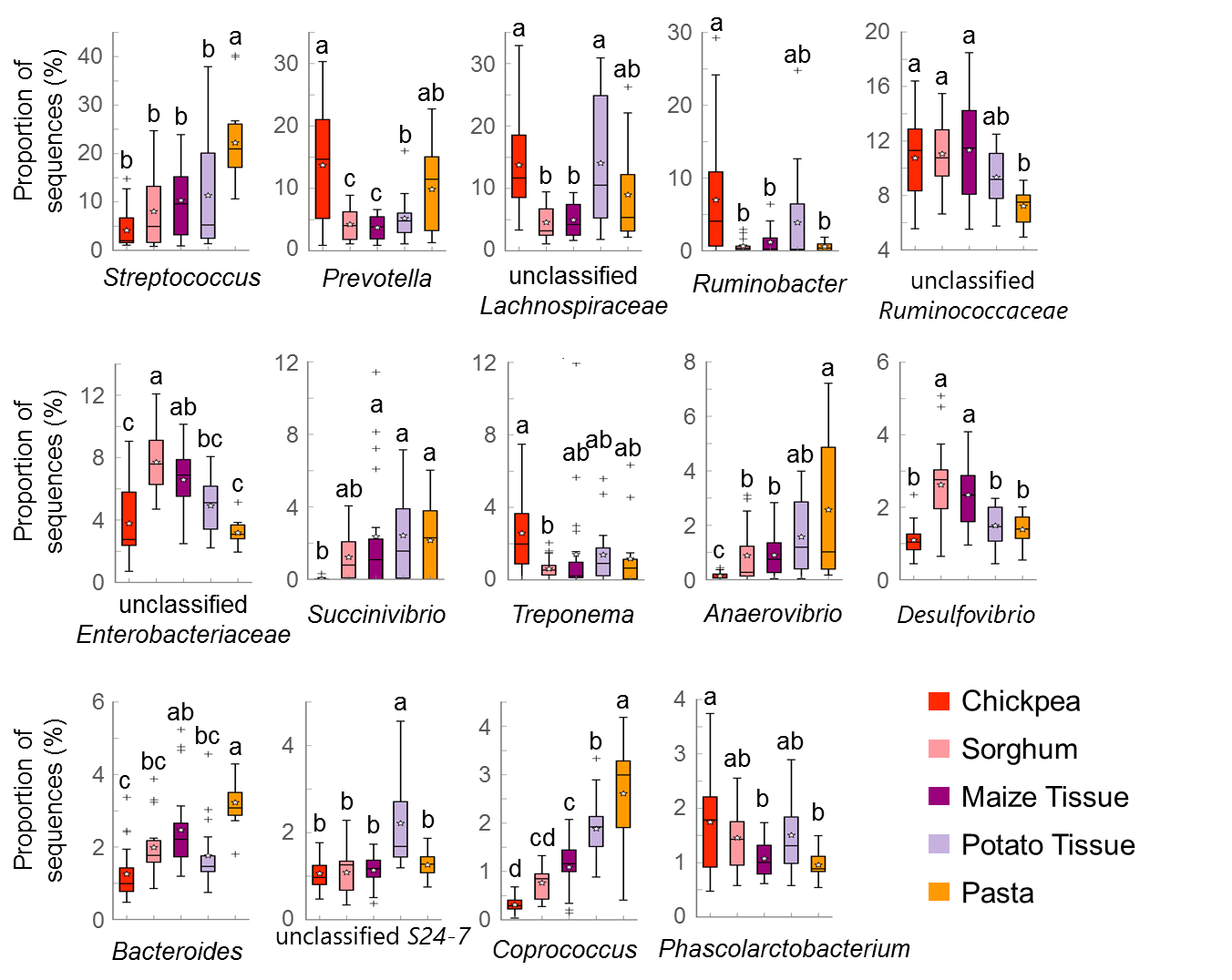


**Figure S3**

Supplement: FIG S3 [file sph003182543sf3.docx]

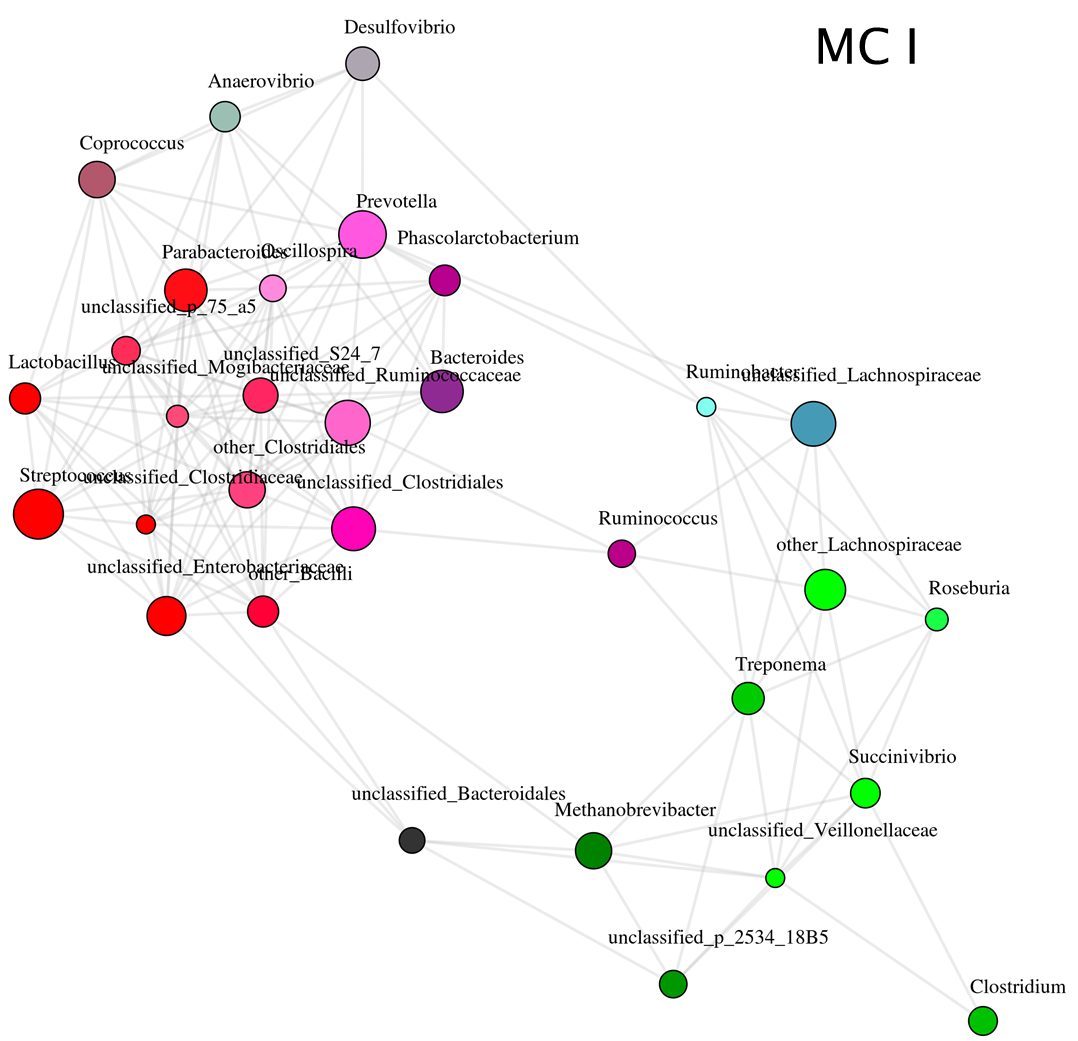


**Figure S4**

Supplement: FIG S4 [file sph003182543sf4.docx]

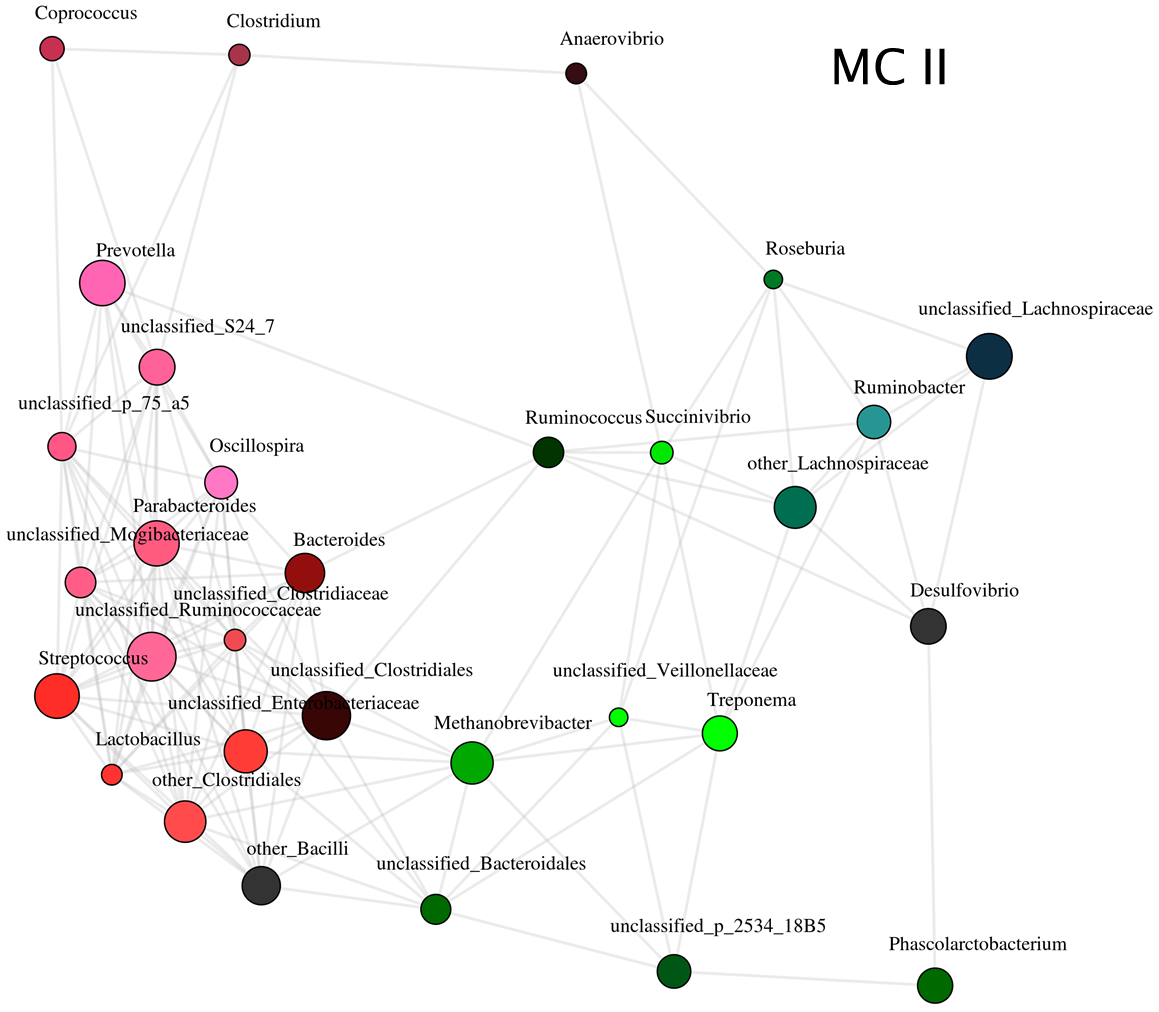


**Figure S5**

Supplement: FIG S5 [file sph003182543sf5.docx]

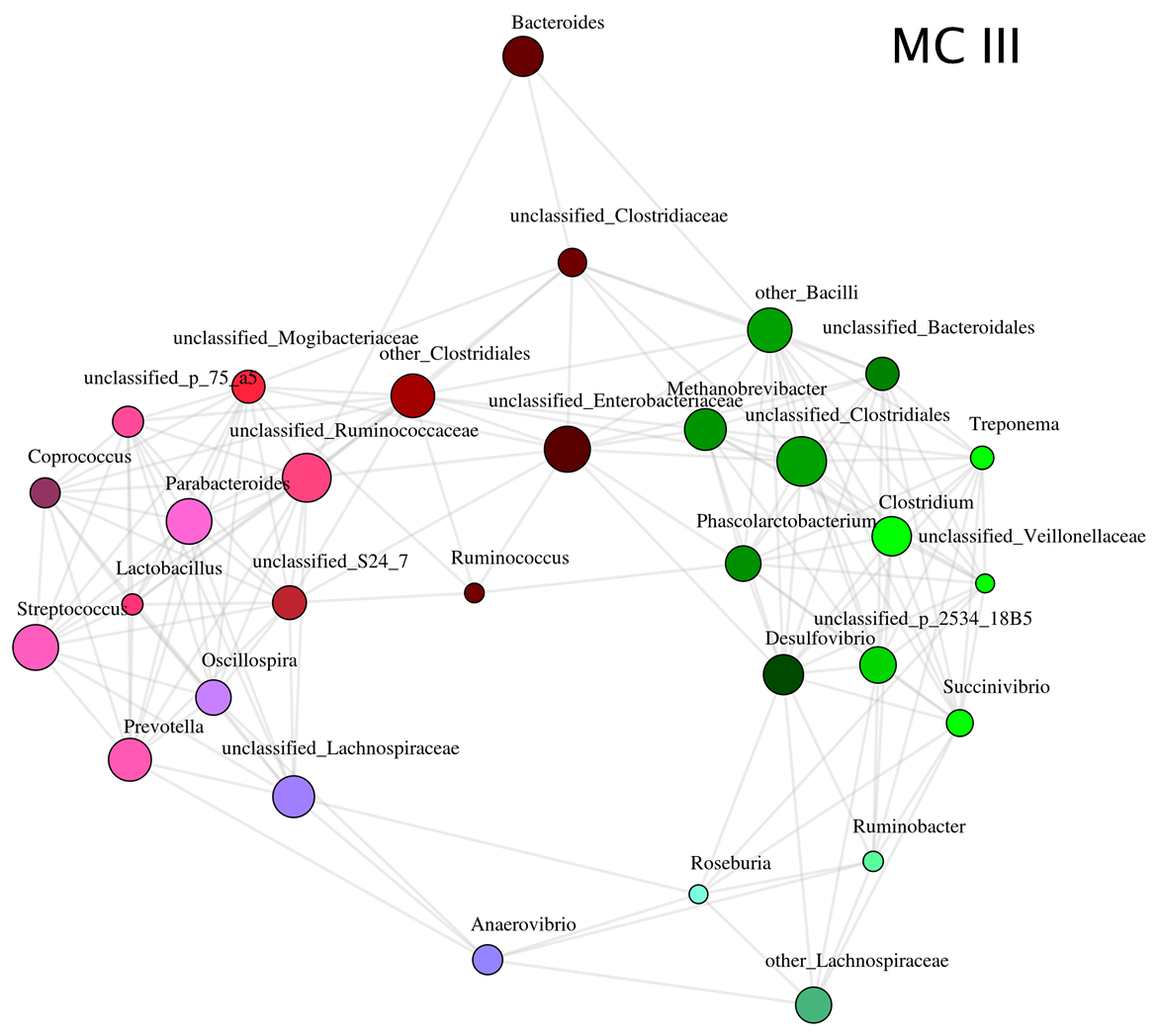


**Figure S6**

Supplement: FIG S6 [file sph003182543sf6.docx]

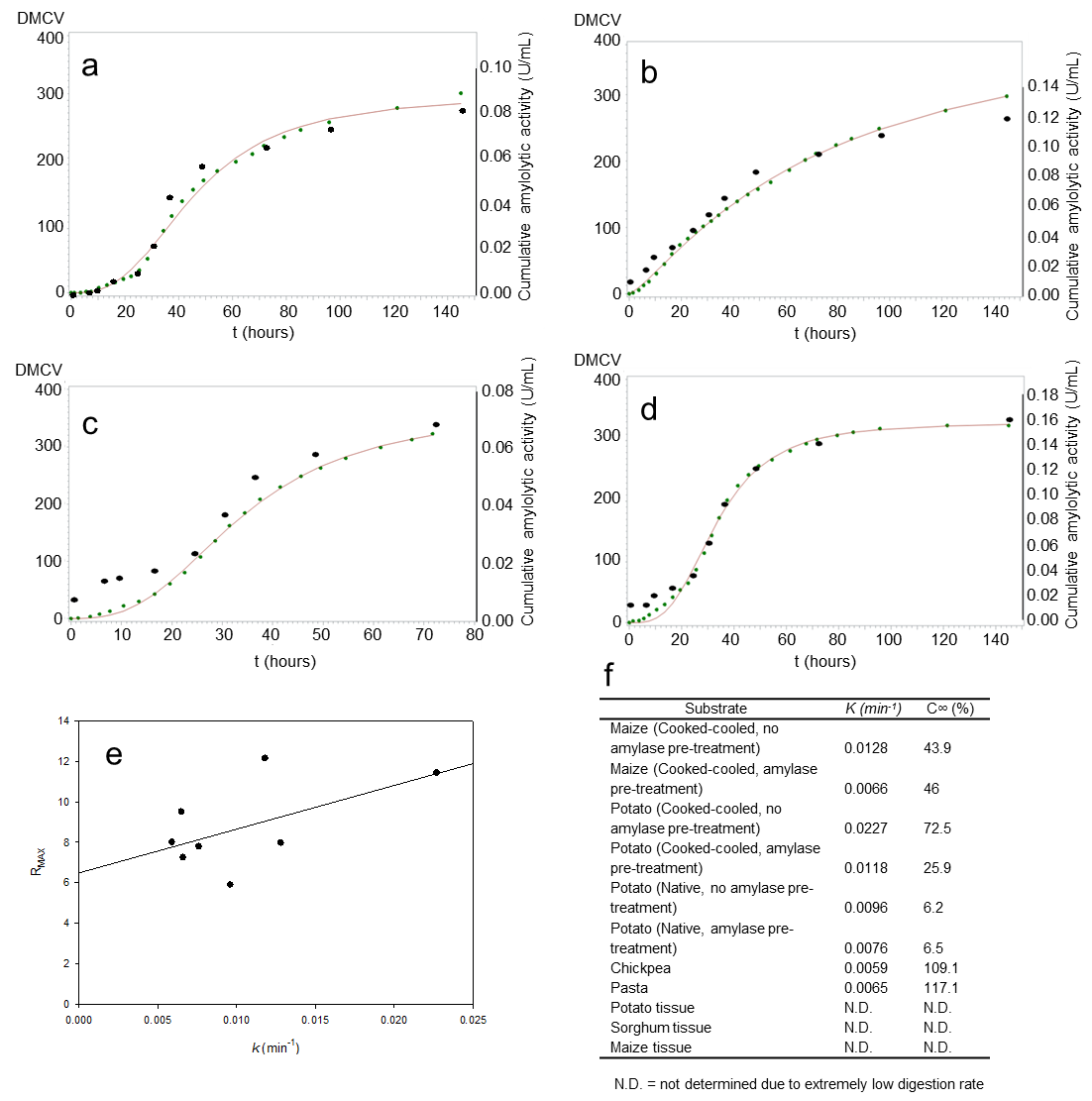


**Figure S7**

Supplement: FIG S7 [file sph003182543sf7.docx]
